# Benchmarking the nutrient composition and labelling practices of commercially produced ready‐to‐eat purées and meals for older infants and young children across seven Southeast Asian countries

**DOI:** 10.1111/mcn.13585

**Published:** 2023-12-13

**Authors:** Eleonora Bassetti, Jessica Blankenship, Jessica M. White, Anzélle Mulder, Diane Threapleton, Alissa M. Pries

**Affiliations:** ^1^ Helen Keller International New York New York USA; ^2^ UNICEF East Asia Pacific Regional Office Bangkok Thailand; ^3^ JB Consultancy Johannesburg South Africa; ^4^ School of Food Science and Nutrition University of Leeds Leeds UK

## Abstract

Commercially produced complementary foods (CPCF), including ready‐to‐eat CPCF purées and meals, are gaining popularity among caregivers of older infants and young children (IYC) as a convenient alternative to home‐prepared foods in low and middle‐income countries. However, there is growing concern regarding the suitability of these products for older IYC, as they can contain high levels of sugar and/or sodium. Given the rapidly evolving market in Southeast Asia, it is crucial to monitor the appropriate composition and promotion of CPCF in the region. This study examined the nutrient composition and labelling practices of CPCF purées and meals sold in 2021 in the capital cities of seven Southeast Asian countries: Phnom Penh (Cambodia), Jakarta (Indonesia), Manila (Philippines), Bangkok (Thailand), Vientiane (Lao PDR), Hanoi (Viet Nam), Kuala Lumpur (Malaysia). The study adapted a nutrient profiling model from the WHO Regional Office for Europe to determine the proportion of products suitable for promotion for older IYC. The proportion of CPCF purées and meals that would require a high sugar front‐of‐pack warning based on the percentage energy from total sugar was also determined. Of the 459 CPCF purées/meals assessed, only 37.7% of the products met all nutrient composition requirements and none met all labelling requirements. In addition, most CPCF purées and meals were identified as having high total sugar content. To ensure that older IYC consume appropriate CPCF products, Southeast Asian countries need to implement and enforce regulations concerning the nutrient composition and labelling practices of CPCF purées and meals.

## Key messages

- Only one‐third of CPCF purées/meals were identified as nutritionally suitable for promotion for older IYC, with performance varying by sub‐category, and over three‐quarters of products contained high levels of total sugar.
- None of the CPCF purées/meals presented fully appropriate labelling practices, with the use of inappropriate claims the most prevalent issue. Further, one‐third of products recommended an age of introduction of less than 6 months.
- To ensure that CPCF purées/meals sold in Southeast Asia have appropriate nutrient composition and labelling practices, it is essential that national standards are in accordance with global guidance and enforced.

## Graphical abstract

This study examined the nutrient composition and labelling practices of commercially produced complementary food (CPCF) purées and meals sold in 2021 in the capital cities of seven Southeast Asian countries. Of the 459 CPCF purées/meals assessed, only one‐third of the products met all nutrient composition requirements and none met all labelling requirements, and most CPCF purées and meals were identified as having high total sugar content.

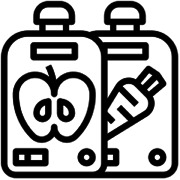

## 1 INTRODUCTION

Optimal infant and young child feeding practices, which include the introduction of safe and adequate complementary foods at 6 months of age, together with continued breastfeeding to 2 years or beyond, are essential for child health, growth and development (Bhutta et al., 2013; De Onis & Branca, 2016; Lobstein et al., 2015; World Health Organization [WHO], 2003). During the complementary feeding period, from 6 months to a child's second birthday, the brain undergoes rapid and dynamic growth and the timing, dose and duration of exposure to specific nutrients has profound impact on lifelong cognitive development (Georgieff et al., 2018). Additionally, the complementary feeding period plays a critical role in shaping the food preferences and eating habits of older infants and young children (older IYC) that may persist into later life (Birch & Doub, 2014; De Cosmi et al., 2017). In Southeast Asia, where the triple burden of malnutrition—characterized by the coexistence of undernutrition, overweight and micronutrient deficiencies within the same individual, household, or population—is a concerning issue, it is essential to ensure adequate diets for older IYC aged 6–36 months (Haddad et al., 2015; UNICEF, 2021; UNICEF/WHO/The World Bank, 2023; WHO, 2021).

Commercially produced complementary foods (CPCF) promoted as suitable for older IYC are increasingly popular as a convenient alternative to home‐prepared foods worldwide, and their market is expanding rapidly in many low and middle‐income countries (Abizari et al., 2017; Champeny et al., 2019; Hadihardjono et al., 2019; Tuan et al., 2017; Zehner et al., 2019). CPCF come in an array of textures, flavours and consistencies from dry cereal‐based porridges and snack foods to ready to eat blended mashes of ingredients or textured meals in jars or pouches. These CPCF purées/meals are the predominant product type sold in mature markets, with sales accounting for roughly 87% of all CPCF in the United States, 88% in France in 2022 and 76% in South Korea in 2020 (GlobalData, 2022). CPCF purées/meals popularity may be attributed to their ease of storage, transport, and use on‐the‐go, making them a practical option for caregivers. According to a recent survey conducted in the United Kingdom, caregivers frequently opted for CPCF purées/meals for their convenience and also because they were perceived as healthy, safe and cost‐effective options for older IYC feeding (Isaacs et al., 2022). CPCF purées/meals are smoother in texture and their labels often display or state a range of foods such as vegetables, fruits, and meats—enabling the perception that they are both healthy and allow an introduction of a range of flavours to older IYC. However, some CPCF purées/meals present undesirable nutrient profiles, with many having high total sugar and/or sodium content (Bridge et al., 2021; Crawley & Westland, 2017; Hutchinson et al., 2021; Koletzko et al., 2018; Maalouf et al., 2017; Sugimoto et al., 2023; Walker & Goran, 2015). In addition, products are frequently promoted as appropriate from 4 months of age, despite the World Health Organization's (WHO) recommendation to exclusively breastfeed for the first 6 months of life (Bridge et al., 2021; Crawley & Westland, 2017; Sweet et al., 2016). Puréed CPCF in squeezable pouches, emerging as the most popular packaging option for CPCF in many countries (Beauregard et al., 2019; Garcia et al., 2020), are convenient for caregivers but present concerns due to the lack of sensory experience of the older IYC seeing, smelling, and touching new foods, interacting with the caregiver and the reduced feeding time, which may negatively impact the development of older IYC eating behaviours (Koletzko et al., 2019).

In 2016, the World Health Assembly approved the WHO Guidance on Ending the Inappropriate Promotion of Foods for Infants and Young Children (WHO Guidance), designed to protect breastfeeding, prevent obesity and chronic diseases, promote a healthy diet and ensure caregivers receive clear and accurate information on older IYC feeding (WHO, 2016, 2017). The WHO Guidance called for the development of nutrient profile models (NPM) to guide decisions about which products are inappropriate for promotion for children aged 6–36 months (WHO, 2017). A draft NPM was created by the WHO Regional Office for Europe (WHO Europe) in 2019 to categorize CPCF, including CPCF purées and meals, according to their nutrient composition and labelling practices (WHO Regional Office for Europe, 2019). The application of the draft WHO Europe NPM has demonstrated that many CPCF have inappropriate nutrient composition and labelling practices, making them unsuitable for promotion for older IYC (Grammatikaki et al., 2021; Hutchinson et al., 2021; Santos et al., 2022; WHO Regional Office for Europe, 2019). In 2022, following a thorough application of the draft WHO Europe NPM in several countries, the WHO Europe released a finalized version of the NPM, known as Nutrient and Promotion Profile Model (WHO Regional Office for Europe, 2022).

At present, in Southeast Asia, there is a lack of national and regional guidance on what constitutes appropriate nutrient composition and package labelling for CPCF purées/meals, and limited evidence is available on the current landscape of CPCF purées/meals sold in the region. Using information provided on the product labels, this study aimed to provide a comprehensive assessment of the nutrient composition and labelling practices of CPCF purées/meals sold in seven Southeast Asia capital cities. The objectives were to assess CPCF purées/meals to determine: (1) the proportion with suitable nutrient composition and labelling practices and (2) the proportion that would require a ‘high sugar’ warning due to excessive total sugar content.

## 2 METHODS

### 2.1 Study design

A cross‐sectional assessment was conducted among CPCF purées/meals purchased between August and December 2021 in the capital cities of Cambodia, Indonesia, Lao People's Democratic Republic (PDR), Malaysia, Philippines, Thailand and Viet Nam. Specifically, purchases were made in Phnom Penh in August, Bangkok in August–September, Hanoi in October and Jakarta, Vientiane and Kuala Lumpur in November. Capital cities were selected due to their expected extensive availability of CPCF, being major urban centres, and because they are where UNICEF national offices are present and national researchers could therefore be engaged. In the Southeast Asian region, Singapore was excluded due to the absence of a UNICEF presence in the country and Myanmar because of a national emergency. Purchased products were benchmarked against nutrient composition and labelling requirements of an adapted version of the WHO Europe NPM for CPCF (adapted NPM for CPCF) to assess their suitability for promotion for children 6–36 months of age. Adaptions were made to the 2019 WHO Europe NPM (WHO Regional Office for Europe, 2019) to align with the finalized Nutrient and Promotion Profile Model released by WHO Europe in 2022 (WHO Regional Office for Europe, 2022) and provide more comprehensive definitions of claims made for CPCF. Results related to other CPCF categories (e.g., dry or instant cereals and finger foods/snacks) from the seven countries are detailed seperately (Bassetti et al., 2023; Pries et al., 2023).

### 2.2 Store sampling and product identification

In Cambodia, Lao PDR, Indonesia, Malaysia, Thailand, and Viet Nam, stores that sold CPCF were identified through a web‐based search and consultation with local experts, and a list of chain and independent retail outlets (including supermarkets, hypermarkets, large grocery stores, baby stores and large pharmacies) was developed. For each chain outlet, the largest physical location was selected, together with all independent retail stores. A web search was conducted to determine if major outlets offered online platforms or physical‐only outlets, and online retailers without physical stores were excluded from the study. Given the significant number of retail outlets (*n* = 67) found in Kuala Lumpur, around half (*n* = 21) of the chain stores per store category and nine independent stores were selected, prioritizing those with the broadest CPCF variety. Store selection for each country was adapted in response to the COVID‐19 pandemic. For safety reasons, only online platforms of physical retailers were visited in Bangkok (*n* = 31), Jakarta (*n* = 25), Kuala Lumpur (*n* = 30), and Hanoi (*n* = 21). In Phnom Penh, both online platforms (*n* = 6) and physical outlets (*n* = 22) were visited. In Vientiane, due to low COVID‐19 incidence and limited online shopping availability, only physical outlets (*n* = 22) were surveyed.

National researchers involved in product purchasing and data extraction underwent a standardized training, remotely administered by a dedicated technical team. The training included the identification of CPCF, the categorization of CPCF, the methodology to search for CPCF in physical and online stores and, finally, step‐wise instructions on how to collect data concerning CPCF in a mobile application called ONA Data app (https://ona.io/home/products/ona-data/features/). The ONA Data App was used as a centralized platform to ensure uniform and efficient data collection.

At each physical store and online platform, the inventory was thoroughly examined for CPCF. Products were classified as CPCF if they met at least one of the following criteria: (1) were recommended for introduction at an age of less than 3 years; (2) were labelled with the words ‘baby’, ‘infant’, ‘toddler’, ‘young child’ or a synonym; (3) had a label with an image of a child who appeared to be younger than 3 years of age or who was feeding with a bottle; or (4) were in any other way presented as being suitable for children under 3 years of age (WHO, 2017). Images of bottles on labels or preparation instructions recommending bottle usage were interpreted as ‘other ways’ suggesting the product's suitability for children under 3 years of age. In every sampled retail outlet, one of each unique CPCF found was purchased and duplicates later deleted from the data set. However, in Viet Nam, due to the notably larger number of CPCF products identified compared to other locations, only one of each unique CPCF was purchased across all retail outlets included in the sample. Products were considered unique if they differed by brand name, subbrand name, descriptive name, age category/recommendation, manufacturer and/or flavour. Single serving and multi‐serving packages, different sizes of multi‐serving packages, and bundles of single‐serving sachets/packages of the same product were considered a single product. Similarly, products that only differed in the type of packaging, such as box or canister, were also classified as a single product. The ONA Data mobile app was employed to record products purchased from both online platforms and physical stores. For online platforms, sections related to infants and young children, such as ‘baby and kids’ or ‘mother and baby’, and the refrigerated/frozen food sections were reviewed for CPCF products. If a search function was available, terms like ‘baby food’, ‘kid food’ or ‘toddler food’ were entered to ensure comprehensive product capture. In physical stores, the search started in the ‘baby and toddler’ area and covered the entire store. If a store operated both online and offline, CPCF products not sold online were procured from the physical location.

The list of CPCF products available in the Philippines was sourced from the Access to Nutrition Initiative (ATNI) CPCF analysis study conducted in 2020 (Access To Nutrition Initiative ATNI, 2021), and the store sampling methodology is explained in Bassetti et al. (Bassetti et al., 2022). The ATNI CPCF list was utilized as a reference to carry out the online CPCF purchasing process, with national researchers referring to the list to search for products, ensuring that the products found online matched the information provided across the company, brand and product name. Products that were no longer available online were excluded from the analysis.

### 2.3 Data capture and management

General product details of each CPCF purchased were recorded using the ONA Data mobile app. Products were excluded from the study if label information was not provided in the official local language of the country or English. The labels of all unique products were photographed, and the images uploaded to ONA Data app. Two national researchers independently conducted data extraction of label information in two steps. In the first step, product names, ingredients and preparation instructions were reviewed to classify products into one of six categories: (1) Dry, powdered and instant cereal/starchy food, (2) Soft–wet spoonable, ready‐to‐eat foods, (3) meals with chunky pieces, (4) Dry finger foods and snacks, (5) Juices and other drinks and (6) other (WHO Regional Office for Europe, 2019). The current study focuses on two of the six categories: ‘soft–wet spoonable, ready‐to‐eat foods’ (purées) and ‘meals with chunky pieces’ (meals). The two categories were further divided into nine subcategories (Table 1), with each subcategory having its own set of requirements for nutrient composition and labelling. In the second step, information on the nutrient composition and labelling practices of the CPCF products were extracted. Label text in either English or the local language was extracted, with priority given to the local language. In cases where only the local language was available, the text was translated to English. Extracted data was exported into Microsoft Excel for analysis. Double data entry was reviewed by a third researcher, and any inconsistencies (e.g., data entry typos, or translation disagreement) was resolved. A 5% error check was carried out against the label images. This error check continued until the error rate was below 5%.

**Table 1:** Categories and subcategories defined as commercially produced complementary food (CPCF) purées/meals of the adapted NPM for CPCF.^a^

Food category	Definition and examples
Soft–wet spoonable, ready‐to‐eat foods	
Dairy‐based desserts and cereal products	Foods with dairy as the largest main ingredient by weight (i.e., greater than the sum of total fruit or total grain ingredients). This may include yogurt, fromage frais, custards, porridge or rice pudding, made with or without other naturally sweet foods such as fresh fruit, fruit juice or dried fruit. Does not contain meat or fish.
Fruit purées	Largest ingredient single or mixed fruit. May contain vegetables, cereals and dairy. Includes any spoonable fruit or fruit‐and‐vegetable purée, high‐fruit breakfast foods (such as fruit‐based breakfast rice/porridge) and desserts (such as apple crumble or fruit‐based baby rice). May include some products labelled as ‘smoothies’, without the addition of juice or water.
Vegetable‐only purées	≥95% single or mixed vegetables or legumes and water combined. Excludes products containing any fruit, or >5% cereals or other ingredients. May include some products labelled as ‘smoothies’ without the addition of fruit or vegetable juice.
Vegetable purées with cereals	Puréed vegetables/legumes, where largest ingredient by weight is vegetables, legumes, cereals or pseudocereals, with >5% cooked weight in cereal (e.g., pasta, rice, barley), or a pseudocereal (such as quinoa, chia, buckwheat). Includes savoury‐type meals with cereals (such as pasta with tomato and courgette) or pseudocereal (such as butternut squash, carrot and quinoa). Does not contain meat or fish. Includes vegetable‐based foods containing cheese, where cheese is not mentioned in the product name.
Puréed meals with cheese	A puréed meal containing cheese, vegetables, starchy carbohydrates, where cheese is mentioned in the name (such as ‘Cheesy pasta with tomato and vegetables’ or ‘Cauliflower cheese’ or ‘Macaroni cheese’). Does not contain meat or fish.
Puréed meals with meat/fish	A puréed meal containing meat or fish in addition to vegetables, and other starchy carbohydrates. May contain other ingredients. Meat or fish is mentioned as first food in product name (such as ‘Tasty fish pie’ or ‘Salmon and pea risotto’ or ‘Hearty beef hotpot’ or ‘Chicken and potato pie’).
Puréed meals without meat/fish	A puréed meal containing meat or fish, vegetables, and starchy carbohydrates, where the fish/meat protein source is not listed as first food in product name (such as ‘Hearty shepherd's pie’, ‘Cottage pie’ or ‘Carrot, potato and lamb hotpot’). May contain other ingredients.
Meals with chunky pieces	
Chunky meal with meat/fish/cheese	A nonpuréed soft meal containing chunky pieces of meat or fish in addition to vegetables, and starchy carbohydrates. May contain other ingredients, such as cheese. Fish or meat is mentioned as first food in product name (such as ‘Tasty fish pie’ or ‘Salmon and pea risotto’ or ‘Heartybeef hotpot’ or ‘Chicken and potato pie’).
Chunky meal with vegetables	A nonpuréed soft meal containing chunky pieces of vegetables, and other starchy carbohydrates. May contain other ingredients such as beans and pulses as sources of protein and iron. May contain meat or fish or cheese not mentioned in the product name.

^a^ WHO Regional Office for Europe (2019). Ending inappropriate promotion of commercially available complementary foods for infants and young children between 6 and 36 months in Europe. https://www.who.int/europe/publications/i/item/WHO-EURO-2019-3590-43349-60813.

### 2.4 Nutrient and labelling profiling methods

CPCF were benchmarked against the adapted NPM for CPCF using label information to determine adherence to nutrient composition and labelling requirements. A CPCF purée/meal was deemed suitable if it met all nutrient composition and labelling requirements.

#### 2.4.1 Nutrient composition requirements

The ingredient list and nutrition information of CPCF purées/meals were assessed against six nutrient composition requirements of the adapted NPM for CPCF: (1) no added sugar/sweeteners (including all mono‐ and disaccharides, syrups, nectars, honey, fruit juices or concentrated/powdered fruit juice (excluding lemon or lime juice), and nonsugar sweeteners), (2) percentage of fruit content, (3) energy density, (4) fat content, (5) protein content and (6) sodium content. The presence of added sugars/sweeteners and the percentage of fruit content were determined from the ingredient lists, while the proportions of energy from fat and protein were calculated using the Atwater factor system. If salt content was listed instead of sodium, the sodium content was determined by dividing the salt content by 2.5. Products were required to meet all six requirements to pass the nutrient composition assessment. Any product without nutrient content information relative to one of the six requirements was noted as having failed that specific requirement. Although not part of the pass/fail assessment, the adapted NPM for CPCF also requires products to provide a front‐of‐pack high sugar warning if the percentage energy from total sugar exceeds the specific threshold for each subcategory. These thresholds are set at ≥40% for dairy‐based foods, ≥30% for fruit purées and vegetable purées, ≥20% for puréed vegetables and cereals, and ≥15% for puréed meals and meals with chunky pieces. Products were assessed based on this additional criterion, and the results reported separately.

#### 2.4.2 Labelling requirements

The adapted NPM for CPCF includes 17 labelling requirements for the CPCF purées/meals category. These requirements cover: the protection and promotion of breastfeeding, claims, product name and ingredient list clarity, messages on products with a spout, and age restrictions on puréed foods. All applicable labelling requirements were required to pass this assessment. Products lacking information for any applicable requirements failed that labelling requirement. Claims were classified into five categories: nonpermitted compositional claims, nutrient content claims, nutrient function claims, disease risk reduction claims, other claims.

### 2.5 Statistical analysis

The software program Stata (version 14.2) was utilized to perform the analysis. Descriptive statistics were calculated and presented using proportions and frequencies.

## 3 RESULTS

### 3.1 Product characteristics

Among all CPCF products, the proportion sold that were purées/meals varied across the seven countries. The highest percentage was in the Philippines (57.1%), followed by Cambodia (40.5%), Viet Nam (32.6%), Thailand (30.6%), Lao PDR (26.3%), Malaysia (14.4%) and Indonesia (12.1%). With the exception of Lao PDR, where the most common subcategory was chunky meals with meat/fish/cheese, across all countries, fruit purées were the predominant CPCF. Fruit purées accounted for 60.9% of CPCF purées/meals in Cambodia, 48.5% in Indonesia, 67.9% in Malaysia, 56.7% in the Philippines, 39.7% in Thailand, and 55.0% in Viet Nam. Table 2 presents the final counts of CPCF purées/meals identified in the seven Southeast Asia countries across subcategories.

**Table 2:** Proportion of CPCF purées/meals passing the relevant nutrient composition requirements of the adapted NPM for CPCF in seven Southeast Asian countries (*n* = 459).^a^

Category	*n*	No added sugar/sweetener^b^	Low/no added fruit	Met energy density requirement^c^	Met sodium requirement	Met total fat requirement	Met protein requirement	Met all relevant nutrient requirements
Dairy‐based desserts and cereal products	42	4.8 (2)	85.7 (36)^d^	97.6 (41)	73.8 (31)^e^	88.1 (37)^f^	NA	0.0 (0)
Fruit purées	245	91.8 (225)	NA	67.8 (166)	91.8 (225)^e^	100.0 (245)^f^	NA	58.8 (144)
Vegetable only purées	25	100.0 (25)	96.0 (24)^g^	24.0 (6)	56.0 (14)^e^	100.0 (25)^f^	NA	12.0 (3)
Vegetable purées with cereals	22	100.0 (22)	59.1 (13)^g^	50.0 (11)	81.8 (18)^e^	100.0 (22)^f^	NA	27.3 (6)
Puréed meals with cheese	9	100.0 (9)	66.7 (6)^d^	88.9 (8)	88.9 (8)^h^	100.0 (9)^i^	100.0 (9)^j^	44.4 (4)
Puréed meals with meat/fish	51	78.4 (40)	82.4 (42)^d^	51.0 (26)	45.1 (23)^k^	82.4 (42)^i^	45.1 (23)^l^	11.8 (6)
Puréed meals without meat/fish	30	80.0 (24)	80.0 (24)^d^	46.7 (14)	56.7 (17)^k^	96.7 (29)^f^	43.3 (13)^m^	10.0 (3)
Chunky meal with meat/fish/cheese	34	94.1 (32)	100.0 (34)^d^	NA	32.4 (11)^k^	97.1 (33)^i^	44.1 (15)^n^	17.7 (6)
Chunky meal with vegetables	1	100.0 (1)	100.0 (1)^d^	NA	100.0 (1)^k^	100.0 (1)^f^	100.0 (1)^j^	100.0 (1)
All products	459	82.8 (380)	84.1 (180)	64.2 (272)	75.8 (348)	96.5 (443)	48.8 (61)	37.7 (173)

Abbreviation: CPCF, commercially produced complementary foods.

^a^ Values are presented as % (*n*).

^b^ Added sugar/sweetener: All mono‐ and disaccharides (including sugars derived from fruits, sugarcane, palms or root vegetables, etc.); all syrups, nectars and honey (including molasses, agave, maple, blossom nectar, malted barley syrup and brown rice syrup, etc.); fruit juices or concentrated/powdered fruit juice, excluding lemon or lime juice (e.g., pear juice, concentrated apple juice or powdered mango juice); and nonsugar sweeteners (such as saccharin, acesulfame, aspartame, sucralose or stevia, etc.).

^c^ Requirement definition: Energy density ≥60 kcal/100 g.

^d^ Requirement definition: ≤5% by weight fruit purée.

^e^ Requirement definition: Sodium <50 mg/100 kcal and <50 mg/100 g.

^f^ Requirement definition: Total fat ≤4.5 g/100 kcals.

^g^ Requirement definition: No added fruit/fruit purée.

^h^ Requirement definition: Sodium <100 mg/100 kcal and 100 mg/100 g.

^i^ Requirement definition: Total fat ≤6 g/100 kcal.

^j^ Requirement definition: Protein ≥3 g/100 kcal.

^k^ Requirement definition: Sodium <50 mg/100 kcal and <50 mg/100 g (or <100 mg/100 kcal and <100 mg/100 g if cheese is listed in front‐of‐pack name).

^l^ Requirement definition: Protein ≥4 g/100 kcal from the named source and protein named as the first food(s) in the product name must be ≥10% by weight of the total product.

^m^ Requirement definition: Protein ≥3 g/100 kcal and protein source mentioned in the product name must be ≥8% by weight of the total product.

^n^ Requirement definition: Protein ≥4 g/100 kcal and protein source mentioned in the product name must be ≥10% by weight of the total product.

### 3.2 Nutrient composition

Of the 459 CPCF purées/meals assessed, approximately one‐third (37.7%, *n* = 173) met all relevant nutrient composition requirements (Table 2). While over half of fruit purées met all composition requirements, products belonging to other subcategories performed less well. Almost all dairy‐based desserts (95.2%, *n* = 40) failed to meet the no added sugars/sweeteners requirement. In addition, despite being typically considered savoury foods, 20.0% (*n* = 6) of puréed meals without meat/fish and 21.6% (*n* = 11) of puréed meals with meat/fish contained at least one added sugar/sweetener. Although most CPCF purées/meals (84.1%, *n* = 180) did not exceed the maximum threshold for added fruit, over one‐third of vegetable purées with cereals and puréed meals with cheese failed to meet this requirement. Additionally, over a third (35.8%, *n* = 187) of all CPCF purées/meals failed to meet the energy density requirement, with only 24.0% (*n* = 6) of vegetable‐only purées and around half of vegetable purées with cereals (50.0%, *n* = 11), puréed meals with meat/fish (51.0%, *n* = 26), and puréed meals without meat/fish (46.7%, *n* = 14) providing minimum energy density. The performance of CPCF purées/meals against the sodium threshold also varied by subcategory. Excessive levels of sodium were found in two‐thirds of chunky meals with meat/fish/cheese (67.6%, *n* = 23), which had a median sodium content of 46 (IQR 23–66) mg per 100 g of product (Table 3). Moreover, approximately half of vegetable‐only purées (44.0%, *n* = 11), puréed meals with meat/fish (54.9%, *n* = 28) and puréed meals without meat/fish (43.3%, *n* = 13) exceeded the sodium threshold. The vast majority (96.5%, *n* = 443) of CPCF purées/meals met the total fat requirement. Most products performed poorly against the protein requirement, which only applied to puréed and chunky meals, with less than half (48.8%, *n* = 61) of these products meeting the requirement.

**Table 3:** Median total sugar, sodium, protein, total fat and saturated fat content per 100 g of CPCF purées/meals.^a^

	Total sugar per 100 g (g)		Sodium per 100 g (mg)		Protein per 100 g (g)		Total fat per 100 g (g)	
Category	*n*	Median [IQR]	*n*	Median [IQR]	*n*	Median [IQR]	*n*	Median [IQR]
Dairy‐based desserts/cereal products	32	8.0 [6.4–10.0]	33	23 [22–40]	42	2.1 [1.7–3.3]	42	3.1 [2.8–3.9]
Fruit purées	227	10.6 [8.8–12.1]	240	6 [2–17]	245	0.9 [0.4–1.0]	245	0.2 [0.0–0.4]
Vegetable only purées	23	3.3 [2.8–4.9]	22	18 [7–27]	25	0.9 [0.9–1.1]	25	0.1 [0.0–0.6]
Vegetable purées with cereals	19	2.8 [2.1–4.6]	22	14 [8–22]	22	1.7 [1.1–1.8]	22	0.4 [0.3–1.0]
Puréed meals with cheese	9	2.8 [1.6–2.8]	9	36 [32–58]	9	2.5 [2.5 – 2.9]	9	2.4 [2.4–2.6]
Puréed meals with meat/fish	38	1.6 [0.8–3.2]	42	23 [15–52]	51	2.9 [2.2–3.7]	42	1.2 [0.6 –1.6]
Puréed meals without meat/fish	23	2.4 [0.8–3.5]	30	28 [20–60]	30	2.7 [2.2–3.2]	30	1.3 [0.8–2.2]
Chunky meals with meat/fish/cheese	26	1.1 [0.7–1.6]	33	46 [23–66]	34	3.0 [2.3–3.7]	33	1.6 [1.2–2.2]
Chunky meals with vegetables	1	1.4	1	22	1	2.8	1	0.9
All products	398	8.4 [3.1–11.0]	432	14 [4–27]	459	1.2 [0.8–2.4]	449	0.4 [0.1–1.5]

Abbreviation: IQR, interquartile range.

^a^ Products without relevant nutrient content declarations on label are excluded.

**Figure 1:**
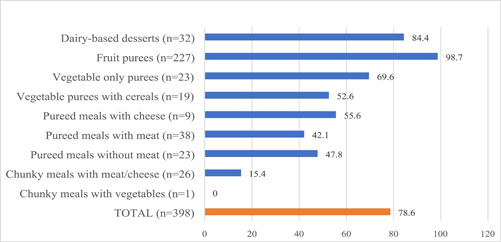
Proportion of products requiring a high sugar front‐of‐pack warning.

Figure 1 shows the percentage of CPCF purées/meals that would warrant a high sugar front‐of‐pack warning on their label based on the adapted NPM for CPCF. Of the 398 (86.7%) products that provided total sugar content on their labels, most (78.6%, *n* = 313) were identified as having high total sugar. This included almost all fruit purées (98.7%), 84.4% of dairy‐based desserts, and over two‐thirds (69.6%) of vegetable only purées. The median total sugar content of fruit purées and dairy‐based desserts was 10.6 (IQR: 8.8–12.1) g and 8.8 (IQR: 6.4–10.0) g per 100 g of product, respectively (Table 3). Additionally, around half of vegetable purées with cereals (52.6%), puréed meals with cheese (55.6%), puréed meals with meat/fish (42.1%) and puréed meals without meat/fish (47.8%) warranted a high sugar front‐of‐pack warning.

### 3.3 Labelling practices

Of the 459 CPCF purées/meals, none passed all labelling requirements (Table 4). Only 39 products (8.5%) passed all five labelling requirements intended to protect and promote breastfeeding. The vast majority of CPCF purées/meals (83.0%, *n* = 381) did not present a message on the importance of continued breastfeeding to 2 years or beyond. Furthermore, around one‐third of the products recommended an age of introduction of less than 6 months (27.4%, *n* = 126) or were marketed as suitable for children under 6 months (28.8%, *n* = 132). The fruit purées and vegetable‐only purées were the subcategories that performed most poorly against these last two requirements. The use of claims on CPCF purées/meals was widely prevalent, with only seven products (1.5%) not including any claims on their labels. Nonpermitted compositional claims (such as ‘contains naturally occurring sugars’, ‘no artificial colours’, ‘no added salt’) were the most commonly displayed type of claim across all subcategories, occurring on almost all products (95.0%, *n* = 436). Furthermore, ‘other claims’ were also particularly prevalent and included ‘easy to swallow texture’, ‘fresh and delicious as mom cooks’, ‘easy to carry anywhere’. Conversely, no products included disease risk reduction claims and only 3.9% (*n* = 18) included nutrition function claims.

**Table 4:** Proportion of CPCF purées/meals passing relevant labelling requirements of the adapted NPM for CPCF in seven Southeast Asian countries (*n* = 459).^a^

	Dairy‐based desserts (*n* = 42)	Fruit purées (*n* = 245)	Vegetable only purées (*n* = 25)	Vegetable purées with cereals (*n* = 22)	Puréed meals with cheese (*n* = 9)	Puréed meals with meat/fish (*n* = 51)	Puréed meals without meat/fish (*n* = 30)	Chunky meals with meat/fish/cheese (*n* = 34)	Chunky meals with vegetables (*n* = 1)	All products (*n* = 459)
Protection and promotion of breastfeeding										
Minimum recommended age of introduction of at least 6 months of age	95.2 (40)	60.8 (149)	64.0 (16)	72.7 (16)	88.9 (8)	86.3 (44)	83.3 (25)	100.0 (34)	100.0 (1)	72.6 (333)
Not marketed as suitable for <6 months	81.0 (34)	62.9 (154)	52.0 (13)	77.3 (17)	66.7 (6)	98.0 (50)	70.0 (21)	91.2 (31)	100.0 (1)	71.2 (327)
Message on importance of breastfeeding ≥2 years	14.3 (6)	20.0 (49)	8.0 (2)	4.6 (1)	44.4 (4)	19.6 (10)	13.3 (4)	5.9 (2)	0.0 (0)	17.0 (78)
Does not suggest superiority or equivalence to breast milk	97.6 (41)	95.1 (233)	100.0 (25)	86.4 (19)	100.0 (9)	100.0 (51)	100.0 (30)	100.0 (34)	100.0 (1)	96.5 (443)
Does not recommend or promote bottle feeding	100.0 (42)	100.0 (245)	100.0 (25)	100.0 (22)	100.0 (9)	100.0 (51)	100.0 (30)	100.0 (34)	100.0 (1)	100.0 (459)
Subtotal	7.1 (3)	11.4 (28)	0.0 (0)	0.0 (0)	11.1 (1)	9.8 (5)	3.3 (1)	2.9 (1)	0.0 (0)	8.5 (39)
Claims										
No nonpermitted compositional claims	19.1 (8)	4.1 (10)	8.0 (2)	0.0 (0)	0.0 (0)	0.0 (0)	10.0 (3)	0.0 (0)	0.0 (0)	5.0 (23)
No nutrient content claims	76.2 (32)	75.5 (185)	88.0 (22)	81.8 (18)	100.0 (9)	58.8 (30)	50.0 (15)	94.1 (32)	100.0 (1)	75.0 (344)
No nutrient function claims	97.6 (41)	98.0 (240)	100.0 (25)	100.0 (22)	100.0 (9)	82.4 (42)	93.3 (28)	97.1 (33)	100.0 (1)	96.1 (441)
No disease risk reduction claims	100.0 (42)	100.0 (245)	100.0 (25)	100.0 (22)	100.0 (9)	100.0 (51)	100.0 (30)	100.0 (34)	100.0 (1)	100.0 (459)
No other claims	28.6 (12)	22.0 (54)	20.0 (5)	9.1 (2)	33.3 (3)	7.8 (4)	26.7 (8)	0.0 (0)	0.0 (0)	19.2 (88)
Subtotal	4.8 (2)	1.6 (4)	4.0 (1)	0.0 (0)	0.0 (0)	0.0 (0)	0.0 (0)	0.0 (0)	0.0 (0)	1.5 (7)
Product name and ingredient list clarity										
Product name reflects ingredients in descending order as per ingredient list	71.4 (30)	64.9 (159)	88.0 (22)	59.1 (13)	33.3 (3)	25.5 (13)	50.0 (15)	50.0 (17)	0.0 (0)	59.3 (272)
Percentage of fruit stated in ingredient list^b^	70.6 (12)	66.8 (163)	NA	53.9 (7)	25.0 (1)	73.3 (11)	50.0 (4)	80.0 (4)	0.0 (0)	65.8 (202)
Percentage of added water stated in ingredient list^c^	11.1 (3)	7.1 (6)	22.7 (5)	25.0 (4)	11.1 (1)	48.7 (19)	33.3 (10)	71.0 (22)	0.0 (0)	26.9 (70)
Percentage of protein stated in ingredient list^d^	NA	NA	NA	NA	NA	92.2 (47)	56.7 (17)	91.2 (31)	100.0 (1)	82.8 (96)
Subtotal	23.8 (10)	25.3 (62)	20.0 (5)	22.7 (5)	0.0 (0)	15.7 (8)	16.7 (5)	41.2 (14)	0.0 (0)	23.5 (108)
Messages on products with a spout										
Product with spout states not to suck from the container^e^	27.8 (5)	15.3 (27)	50.0 (2)	7.1 (1)	0.0 (0)	40.9 (9)	0.0 (0)	NA	NA	17.6 (44)
Product with spout warns that cap is a choking hazard^e^	83.3 (15)	77.3 (136)	25.0 (1)	92.9 (13)	100.0 (6)	50.0 (11)	80.0 (8)	NA	NA	76.0 (190)
Subtotal	27.8 (5)	5.7 (10)	0.0 (0)	0.0 (1)	0.0 (0)	13.6 (3)	0.0 (0)	NA	NA	7.2 (18)
Age restriction on puréed products										
Maximum recommended age of use of 12 months^f^	0.0 (0)	0.0 (0)	4.0 (1)	0.0 (0)	11.1 (1)	0.0 (0)	0.0 (0)	NA	NA	0.5 (2)
Met all relevant labelling requirements	0.0 (0)	0.0 (0)	0.0 (0)	0.0 (0)	0.0 (0)	0.0 (0)	0.0 (0)	0.0 (0)	0.0 (0)	0.0 (0)

Abbreviation: CPCF, commercially produced complementary foods.

^a^ Values are presented as % (*n*).

^b^ Question applicable to 307 products containing fruit (Dairy‐based desserts *n* = 17; Fruit purées *n* = 244; Vegetable purées with cereals *n* = 13; Puréed meals with cheese *n* = 4; Puréed meals with meat/fish *n* = 15; Puréed meals without meat/fish *n* = 8; Chunky meals with meat/fish/cheese *n* = 5; Chunky meals with vegetables *n* = 1).

^c^ Question applicable to 260 products containing added water (Dairy‐based desserts *n* = 27; Fruit purées *n* = 85; Vegetable only purées *n* = 22; Vegetable purées with cereals *n* = 16; Puréed meals with cheese *n* = 9; Puréed meals with meat/fish *n* = 39; Puréed meals without meat/fish *n* = 30; Chunky meals with meat/fish/cheese *n* = 31; Chunky meals with vegetables *n* = 1).

^d^ Question applicable Puréed meals with meat/fish (*n* = 51), Puréed meals without meat/fish (*n* = 30), Chunky meals with meat/fish/cheese (*n* = 34) and Chunky meals with vegetables (*n* = 1).

^e^ A total of 250 products with spouts were assessed against this question (Dairy‐based desserts *n* = 18; Fruit purées *n* = 176; Vegetable only purées *n* = 4; Vegetable purées with cereals *n* = 14; Puréed meals with cheese *n* = 6; Puréed meals with meat/fish *n* = 22; Puréed meals without meat/fish *n* = 10).

^f^ Question applicable to 424 puréed products (Dairy‐based desserts *n* = 42; Fruit purées *n* = 245; Vegetable only purées *n* = 25; Vegetable purées with cereals *n* = 22; Puréed meals with cheese *n* = 9; Puréed meals with meat/fish *n* = 51; Puréed meals without meat/fish *n* = 30).

Less than a quarter (24.4%, *n* = 112) of CPCF purées/meals met all four labelling requirements relative to product name and ingredient list clarity. Approximately two‐thirds of the products had a name that reflected their ingredients in descending order as per the ingredient list (59.3%, *n* = 272). Only 26.9% (*n* = 70) of the products included the percentage of added water in their ingredient lists, with fruit purées performing most poorly against this requirement (7.1%, *n* = 6). In addition, nearly one‐third (34.2%, *n* = 105) of the products that contained fruit did not state the percentage of fruit in the ingredients list. Despite most relevant products including protein by percentage weight in their ingredient lists, 43.3% (*n* = 13) of puréed meals without meat/fish failed to meet this requirement. Among products with a spout, most (76.0%, *n* = 190) presented a warning that the cap is a choking hazard. However, only 17.6% (*n* = 44) of the products with a spout included the message ‘not to suck from the container’. Finally, almost all puréed products (99.5%, *n* = 422) did not include a maximum recommended age of use of 12 months on their labels.

## 4 DISCUSSION

This study benchmarked CPCF purées/meals sold in seven Southeast Asia countries against nutrient composition and labelling requirements in an adapted NPM for CPCF. Of the 459 CPCF purées/meals assessed, just over one‐third met all nutrient composition requirements, and no product met all labelling requirements. Therefore, none of the CPCF purées/meals were deemed suitable for promotion for older IYC.

Approximately 17.2% of purées/meals contained added sugars and/or sweeteners such as sugar, honey or fruit juice. While most products did not contain any added sugars, the vast majority had a high total sugar content, that would require a front‐of‐pack ‘high sugar’ warning. Fruit purées and vegetable‐only purées were the most likely to have excessive levels of total sugars. These findings align with previous research showing that despite CPCF purées/meals often being free from added sugars/sweeteners, they commonly contain high sugar levels (Crawley & Westland, 2017; Dunford et al., 2015; Grammatikaki et al., 2021; Hutchinson et al., 2021; Marais et al., 2019; WHO Regional Office for Europe, 2019). A 2016 study of CPCF purées/meals sold in the United Kingdom found a high proportion of fruit and sweet‐tasting vegetables in the ingredients list, contributing to a high total sugar content, while bitter/less sweet vegetables were rarely used (Garcia et al., 2016). Moreover, among CPCF available in Europe, most ‘fruit products, desserts and yoghurts’ claimed to have no added sugars, however almost 35% of them contained free sugars, fruit and vegetable purées and powders as added ingredients. High sugar content of CPCF purées/meals obtained from fruits and vegetables may generally be considered healthier by consumers as it is primarily intrinsic and not from added sugars. However, the intense puréeing process used to make these products releases intrinsic sugars from the cell walls of fruit and vegetables, resulting in readily available free sugars, which have the same effect as other forms of added sugar (WHO Regional Office for Europe, 2022).

Furthermore, a notable number of products that appear to be savoury options, such as vegetable purées with cereals and puréed meals with cheese, exceeded the maximum amount of added fruit defined in the adapted NPM for CPCF. Similarly, a study in the United Kingdom and Denmark found that sugar content of many savoury CPCF meals was derived from the addition of fruit purées, with many containing over 15% energy from total sugars (WHO Regional Office for Europe, 2019). Garcia et al. noted that savoury CPCF purées/meals with a higher fruit content have significantly higher sugar content, suggesting that the addition of fruit to these products may be primarily to function as a sweetening agent, rather than as an opportunity to introduce older IYC to new fruit and vegetable flavours (Garcia et al., 2016). As children naturally prefer sweet taste and tend to reject bitter and sour taste, it is essential that they are exposed to a variety of flavours to accept and consume a wide range of foods (Mennella et al., 2016; Ventura & Worobey, 2013). The presence of added fruit in CPCF purées/meals can enhance palatability of these products and mask bitter flavours of other ingredients. This may lead caregivers to believe they are introducing new tastes to their children, while instead reinforcing preferences for sweet tastes. While fruits are valuable sources of fibre, vitamins, and minerals, and consumption of fruits is a key recommendation for older IYC, it is recommended that total sugar content of CPCF purées/meals, including that from fruits and sweet vegetables, be considered when evaluating these products. The predominance of excessively sweet CPCF purées/meals available in Southeast Asia is concerning. Excessive sugar intake in early childhood is linked to later health status, including weight gain and dental caries (Breda et al., 2019; Koletzko et al., 2018). Moreover, frequent consumption of sweet products early in life may increase the likelihood of high consumption of such foods later in life (De Cosmi et al., 2017; Foterek et al., 2016).

A minimum energy density is required for some CPCF purées/meals to ensure that they provide adequate energy content to meet the high needs of older IYC and are not predominantly composed of water (WHO Regional Office for Europe, 2019). Over a third of CPCF purées/meals failed to meet the minimum energy density requirement, with vegetable‐only purées performing most poorly. Similarly, a substantial proportion of CPCF purées/meals with low energy density has been observed across Europe (WHO Regional Office for Europe, 2019), and in Australia (Scully et al., 2023). Moreover, a review of CPCF available in the United Kingdom noted that the water content of many CPCF was greater than homemade foods (Crawley & Westland, 2017). CPCF purées/meals with low energy density can be problematic, since older IYC's small gastric capacity means they consume relatively small amounts at mealtimes (Kathryn G., 2013), making essential that energy density comes from nutrient‐rich sources (WHO, 2003). To increase the energy density, manufacturers should be encouraged to reduce, and declare, the proportion of added water content of products.

The adapted NPM for CPCF also stipulates maximum permitted sodium content to prevent older IYC becoming accustomed to a diet high in salt. While most CPCF purées/meals did not exceed the maximum sodium requirement, over half of chunky meals with meat/fish/cheese and puréed meals with meat/fish failed this requirement. Similar observations were made for these product categories available in Brazil (De Araújo et al., 2023). High sodium intake in early life is correlated with high blood pressure in childhood and adulthood, increasing their risk for hypertension and its related morbidities (Lava et al., 2015). The complementary feeding period is important for setting taste preferences and infant attitudes towards foods, and evidence suggests that nutritional habits formed in infancy track into childhood and beyond (De Cosmi et al., 2017). Thus, decreasing early exposure to salty tasting foods is recommended to reduce salt intake later in life (Lava et al., 2015; Liem, 2017).

Animal‐source protein minimums (meat, offal, poultry or fish) in the adapted NPM for CPCF guarantee that any named protein sources are not ‘bulked’ or replaced by cheaper alternatives and that meal products provide a minimum amount of protein. Our finding that more than half of CPCF purées/meals listing a protein source on the front of pack name failed to meet the protein requirement mirrored findings from the United Kingdom (Crawley & Westland, 2017). It is noteworthy that CPCF meals are specifically designed to serve as main food options; as such they should be nutritionally complete, presenting sufficient protein and energy content. CPCF protein content is typically lower than comparable homemade recipes, which may contribute to CPCF having a lower nutrient density, including iron and zinc density (Crawley & Westland, 2017). While diets of older IYC are typically adequate in total protein, even those that are primarily cereal based (Lutter et al., 2021), this age group requires high‐quality sources of protein for optimal growth and development, particularly animal‐source proteins, as these can leverage critical nutrients, including iron and zinc, in older IYC diets (Krebs et al., 2014; WHO Regional Office for Europe, 2022).

Labelling practices of CPCF products can be highly influential, either providing educational messages that support optimal feeding or misleading messages that result in suboptimal practices (Walls et al., 2023). Almost all CPCF purées/meals failed the set of five labelling requirements intended to protect and promote breastfeeding, primarily because they did not present a message on the importance of continued breastfeeding to 2 years or beyond. Moreover, almost one‐third of the products presented a recommended age of introduction of less than 6 months, and 28.8% were marketed as suitable for children under 6 months (presenting images or texts associated with infants under 6 months). According to WHO global guidance, exclusive breastfeeding is recommended for the first 6 months of life, followed by the introduction of appropriate complementary foods from 6 months of age together with continued breastfeeding to 2 years of age or beyond (WHO, 2003). Labels should support these practices to prevent early food introduction and/or displacing breast milk intake (Smith et al., 2015). Breastfeeding practices in Southeast Asia are suboptimal, with less than half of infants under 6 months exclusively breastfed in Lao PDR, Malaysia and Viet Nam, and only 14% in Thailand (UNICEF, 2023). Additionally, less than a quarter of children are still breastfed in the second year of life in Thailand and Viet Nam, and less than half in Lao PDR and Malaysia (UNICEF, 2023).

Claims on CPCF purées/meals labels were widely prevalent, with compositional claims being the most commonly displayed. Extensive use of claims has been also noted on CPCF products available in the United Kingdom (Garcia et al., 2022), Australia (Simmonds et al., 2021) and Taiwan (Koo et al., 2018). Claims tend to emphasize the natural and organic nature of CPCF purées/meals, as well as the absence of artificial additions or other components often considered by consumers as less desirable, such as added sugar and salt. Products that include claims are likely to attract caregivers who perceive products as more healthy and nutritious, leading to the development of brand‐loyalty and idealisation of the product, while also implying superiority over other foods (Harris et al., 2011; Isaacs et al., 2022). A survey conducted by Public Health England found that parents assume nutrient claims ‘no added sugar or salt’ mean the product is healthy and appropriate for older IYC (Public Health England PHE, 2019). Such claims can be misleading, especially when placed on products with undesirable qualities, such as high total sugar content. Previous research has found that a significantly greater proportion of products with ‘no added sugar’ claims were classified as having high sugar content as compared to those without such claims (Garcia et al., 2022; Grammatikaki et al., 2021; Koo et al., 2018). Codex Alimentarius Guidelines on Formulated Complementary Foods for Older Infants and Young Children and WHO Europe NPM discourage the use of nutrition and health claims on foods marketed for older IYC to prevent the promotion of CPCF from contradicting public health messages or undermining caregivers' trust in homemade foods, or implying that certain product features are beneficial (Codex Alimentarius, 2013; WHO Regional Office for Europe, 2022).

Almost all puréed products did not include a maximum recommended age of use of 12 months on their labels. This upper age restriction was set because once teeth emerge and older infants develop fine and gross motor skills, they no longer require puréed foods and should progress to consuming age‐appropriate regular or family‐style foods (WHO Regional Office for Europe, 2019). Moreover, consumption of puréed products by children over 12 months could lead to overeating, as food can be rapidly swallowed without chewing, and ongoing exposure to purées, with high liberated sugar content is a concern for oral health (Crawley & Westland, 2017).

Caregivers of older IYC may perceive CPCF purées/meals are healthy, safe and beneficial options for a child's diet. Research conducted in the United Kingdom reported that parents who chose CPCF purées/meals trusted these products to contain appropriate levels of salt and sugar, as well as a suitable texture, while avoiding ingredients that older IYC should not consume (Isaacs et al., 2022). However, our findings demonstrate that most CPCF purées/meals are not suitable for promotion for older IYC due to excessive levels of sugar and sodium, low energy‐density and inappropriate labelling practices. Given the growing demand and supply of CPCF purées/meals in Southeast Asia, it is crucial to establish strong and enforceable regulations to ensure the appropriate nutrient composition and labelling practices of these products. While Codex standards provide global guidance for developing national regulations on CPCF purées/meals, the ‘WHO Guidance on Ending the Inappropriate Promotion of Foods for Infants and Young Children’ notes that current Codex standards are insufficient to determine the appropriateness of contemporary commercial products marketed for older IYC, and calls for new or updated Codex standards (WHO, 2017). In Southeast Asia, current national regulations are heterogeneous: for both nutrient composition and labelling requirements, with most legally binding measures only partially aligned with Codex, and do not comply with WHO Guidance (Blankenship et al., 2023). To ensure older IYC receive nutritionally appropriate products that are labelled to properly inform caregivers, current national regulations must be enforced and a new or revised national standard for CPCF purées/meals, aligned with updated global guidance, must be established.

The study has some limitations. Even though data were obtained on a large number of commercial CPCF purées/meals (*n* = 459) across seven countries in Southeast Asia through primary data collection, product identification was limited to capital cities of each country. Therefore, the sample of CPCF purées/meals obtained may not be exhaustive of all products available in the market in these countries. Moreover, due to COVID‐19 pandemic, in Indonesia, Malaysia, Thailand and Viet Nam, the search for CPCF was restricted to online platforms. This meant that store categories without online platforms, such as pharmacies, independent supermarkets and baby stores, were not sampled. However, as most major CPCF retailers in these countries operated online, this approach is believed to have had a limited influence on the results. Finally, the sample of CPCF assessed was collected in 2021 and may not fully capture the current market landscape, as products may have been altered, discontinued, or replaced, and new CPCF may have been introduced.

## AUTHOR CONTRIBUTIONS

Jessica Blankenship and Alissa M. Pries designed the study. Anzélle Mulder and Alissa M. Pries conducted the data management. Alissa M. Pries analysed the data. Eleonora Bassetti wrote the paper, and all authors provided substantial review.

## CONFLICT OF INTEREST STATEMENT

The authors declare no conflict of interest.

## Data Availability

The data that support the findings of this study are available from the corresponding author upon reasonable request.
